# Commensal HPVs Have Evolved to Be More Immunogenic Compared with High-Risk α-HPVs

**DOI:** 10.3390/vaccines12070749

**Published:** 2024-07-07

**Authors:** Ranya Guennoun, Anton Alyakin, Hiroshi Higuchi, Shadmehr Demehri

**Affiliations:** 1 Center for Cancer Immunology and Cutaneous Biology Research Center, Department of Dermatology and Krantz Family Center for Cancer Research, Massachusetts General Hospital and Harvard Medical School, Boston, MA 02129, USA; 2 Department of Dermatology, Massachusetts General Hospital and Harvard Medical School, Boston, MA 02114, USA; 3 Washington University School of Medicine, St. Louis, MO 63110, USA; 4 Department of Neurosurgery, NYU Langone Health, New York, NY 10016, USA

**Keywords:** cutaneotropic human papillomavirus, immunogenicity, human leukocyte antigen, skin virome, T cell immunity

## Abstract

Commensal human papillomaviruses (HPVs) are responsible for persistent asymptomatic infection in the human population by maintaining low levels of the episomal genome in the stratified epithelia. Herein, we examined the immunogenicity of cutaneotropic HPVs that are commonly found in the skin. Using an in silico platform to determine human leukocyte antigen (HLA)–peptide complex binding affinity, we observed that early genes of cutaneotropic HPV types within the same species can generate multiple conserved, homologous peptides that bind with high affinity to HLA class I alleles. Interestingly, we discovered that commensal β, γ, μ, and ν HPVs contain significantly more immunogenic peptides compared with α-HPVs, which include high-risk, oncogenic HPV types. Our findings indicate that commensal HPV proteins have evolved to generate peptides that better complement their host’s HLA repertoire. Promoting higher control by host T cell immunity in this way could be a mechanism by which HPVs achieve widespread asymptomatic colonization in humans. This work supports the role of commensal HPVs as immunogenic targets within epithelial cells, which may contribute to the immune regulation of the skin and mucosa.

## 1. Introduction

Human papillomavirus (HPV) is a non-enveloped, icosahedral, double-stranded, circular DNA virus that can infect cutaneous and mucosal epithelia [1]. There are approximately 200 different HPV types, which are divided into five genera: α, β, γ, μ, and ν. HPV taxonomy is based on the nucleotide sequence of the most conserved gene in the HPV genome, L1, which encodes for the capsid protein. The original classification of HPVs establishes that if two HPVs have more than 70% nucleotide homology in the L1 gene, they belong to the same type, whereas if they share more than 60% nucleotide homology, they belong to the same species [2]. More recently, it has been determined that a novel HPV type shares less than 90% similarity to any other existing HPV type [3,4,5]. Despite multiple studies on their potential link to carcinogenesis, commensal HPVs belonging to β and γ genera have not been shown to directly cause cancer in skin or mucosal sites. A “hit-and-run hypothesis” suggests that β-HPVs are involved in the initiation of cutaneous squamous cell carcinomas (cSCC) but become dispensable in the progression of carcinogenesis. In fact, β-HPVs have been found to be more abundant in cSCC precursors, actinic keratoses (AK) than in cSCCs [6]. Interestingly, we have found that mouse papillomavirus (MmuPV1) infection plays a protective role against cSCC development in immunocompetent hosts [7]. Our research has demonstrated that T cell immunity to commensal papillomaviruses suppresses skin cancer development in immunocompetent hosts, and the loss of this immunity, rather than the oncogenic effect of cutaneotropic HPVs, causes the increased risk of skin cancer in immunosuppressed patients [7]. Consistent with these findings, β-HPV E7 peptides activate skin resident CD8^+^ T cells in the normal skin of immunocompetent individuals [7]. Cutaneous virome research is a growing field, but there are limited data on the viral populations that colonize the human skin. High-throughput sequencing of skin swab samples from healthy individuals as well as immunosuppressed patients such as organ transplant recipients (OTRs) and DOCK8 deficiency shows that even across individual samples, there is great diversity in HPV types [8,9]. Immunosurveillance prevents an outburst of cutaneous warts caused by HPVs by keeping these viruses at bay with low-copy replication in the basal layer. When this immunosurveillance is lacking, such as in OTRs [10], WHIM syndrome patients [11], DOCK8-deficient patients [12], epidermodysplasia verruciformis patients [1], and other inborn errors of immunity [13,14], susceptibility to warts and expansion of the viral flora, including HPVs, become evident.

Understanding the relationship between commensal HPVs and tissue homeostasis requires the study of the interactions between HPV and host immunity. However, this understanding is very limited compared to the well-studied high-risk α-HPVs [15]. In this study, we used NetMHCpan 4.1 as a sequence-based platform for the following peptide: human leukocyte antigen (HLA) class I binding affinity predictions. It is a well-established, experimentally validated platform with high accuracy that uses artificial neural networks (ANNs) and has been used in the study of peptide vaccine design, immunogenicity predictions, and host immunity to pathogens [16]. Using an in silico-based approach, we sought to determine the immunogenicity of commensal HPVs compared to high-risk α-HPVs. Interestingly, we found that β- and γ-HPVs have evolved to generate significantly more immunogenic peptides compared with α-HPVs, including high-risk α-HPV types.

## 2. Materials and Methods

### 2.1. Sequences and HLAs

HPV sequences were obtained from the National Institute of Allergy and Infection Diseases “PaVE: The Papillomavirus Episteme” database. HPV sequences are included in Supplementary Material Table S6 “HPV Sequences.csv”. Cutaneotropic HPV types found in common or recalcitrant warts of OTRs, as well as α-HPVs were selected for this study.

We chose 18 HLA class I alleles for our study, 6 HLA-A, 6 HLA-B, and 6 HLA-C, that had the highest frequency in the Caucasian-identifying US population. Frequencies and HLAs are listed in Table S1. These were obtained from the “Be The Match” HLA frequency registry (https://bioinformatics.bethematchclinical.org/hla-resources/haplotype-frequencies/high-resolution-hla-alleles-and-haplotypes-in-the-us-population/ (accessed on 1 January 2022)).

### 2.2. HPV Protein Homology

For each of the four early HPV proteins (E1, E2, E6, and E7), we performed pairwise alignments between all possible pairs of viral protein sequences from two different HPV types (excluding comparisons within the same virus), resulting in a total of 1225 alignments (50 sequences from each virus, giving 50 × 49/2 alignments). The alignments were obtained using the Needleman–Wunsch dynamic programming algorithm implemented in Biopython 1.79 [17]. We used the BLOSUM62 substitution matrix [18,19,20,21,22] and set the gap existence penalty to −11 and the gap extension penalty to −1. The percent identity between two sequences was calculated as the number of identical amino acids in the aligned regions.

### 2.3. HPV Immunogenic Peptide Predictions

To identify immunogenic peptides that bind to at least one of the 18 HLA complexes, we utilized the local Darwin version of NetMHCpan—4.1 [16]. For ease of use, we employed a Python wrapper called mhctools 1.8.1, available at https://github.com/openvax/mhctools (accessed on 1 January 2022). The list of immunogenic peptides was sorted based on their binding affinities, and peptides with affinities stronger than 500 nM were considered. To avoid duplication, any peptides that showed strong binding affinities to multiple HLA complexes were included only once. Although a more recent platform for peptide–HLA binding affinity predictions exists [23] that takes into consideration structural interactions in addition to sequence information, we found NetMHCpan to be sufficient for our comparisons of relative binding affinities of selected HPV proteins.

### 2.4. HPV Immunogenic Epitope Clustering

For each early HPV protein analyzed (E1, E2, E6, and E7), a peptide match was defined as having >88% identity, meaning at least 8 out of 9 amino acids were identical and in the same positions. The total number of matches was tallied. Our methodology allowed for the same peptide from one virus to have multiple matches in other viruses, and each match was counted independently.

### 2.5. Phylogenetic Tree Construction

To construct the phylogenetic tree, we used the Multiple Sequence Comparison by Log-Expectation (MUSCLE) tool, available at https://www.ebi.ac.uk/Tools/msa/muscle/ (accessed on 1 January 2022), for multiple sequence alignment. Next, we computed the distance between sequences using the percent identity metric. We then constructed the phylogenetic tree using the neighbor-joining algorithm available in Biopython’s Phylo module. The neighbor-joining algorithm produces an unrooted tree, but for the sake of clarity, we rooted our tree. Additionally, we plotted the distance from the hypothetical common shared ancestor (root) against the number of predicted immunogenic peptides. If the same peptide was predicted to be immunogenic with multiple HLA complexes, it was counted only once. The data and code used for this analysis can be found at https://github.com/alyakin314/hpv-immunogenicity.

## 3. Results

### 3.1. Early HPV Proteins Have High Amino Acid Sequence Homology at the Species Level

**Figure 1:**
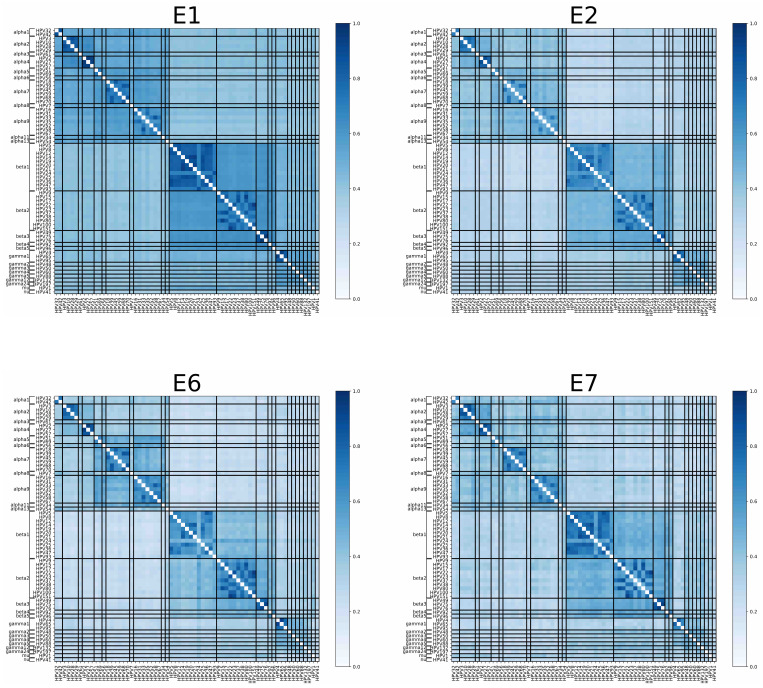
Heatmap of percent amino acid sequence homology between early proteins of selected HPV types. Using Biopython implementation of the Needleman–Wunsch dynamic programming algorithm, we obtained global pairwise alignment between two sequences to depict protein homology between the HPV types we have selected. Each heatmap represents one early protein (E1, E2, E6, and E7). Each small square on the four panels depicts the percent homology between two HPV types. Darker shades of blue represent a higher percentage of homology. For each protein, there were statistically significantly higher homologies when comparing in-species HPV pairs to out-of-species HPV pairs (see text for *p*-values).

HPV early proteins are required for viral maintenance and are expressed in the basal epithelial layer during persistent infection [24,25]. To determine the amino acid sequence homology between HPV early proteins, E1, E2, E6, and E7, we examined 67 HPV types, which include cutaneous and mucosal types commonly found in the skin and warts, as well as high-risk HPVs (Table S2). Using the Biopython implementation of the Needleman–Wunsch dynamic programming algorithm, we obtained global pairwise alignment between sequences to depict protein homology between the HPV types (Figure 1). There was a significantly higher homology between viruses within each species compared to viruses that belong to different species for E1 (*p*-value of 2.58 × 10^−99^), E2 (*p*-value of 9.87 × 10^−100^), E6 (*p*-value of 1.43 × 10^−97^), and E7 (*p*-value of 1.83 × 10^−98^) (Table S3).

### 3.2. Early HPV Genes within a Species Generate Multiple Conserved, Immunogenic Peptides

**Figure 2:**
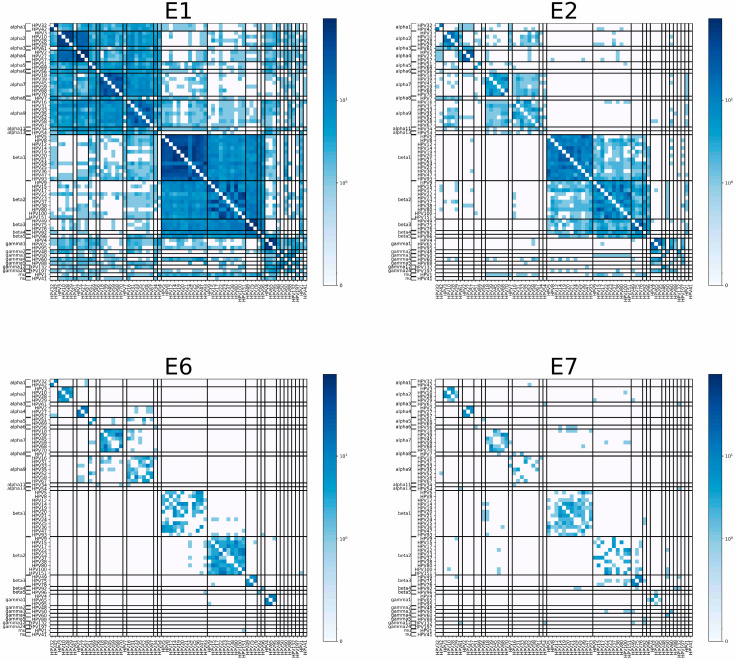
Heatmap of conserved peptide matches between HPV types that bind with high affinity to HLA class I haplotypes. Using the NetMHCpan software (version 4.1), we obtained a list of 9mer peptides that bind to our selected HLAs with less than 500 nM binding affinity. We defined a shared immunogenic peptide as one with at least 8/9 or 9/9 identical amino acids, where order matters. Darker shades of blue represent a higher number of conserved peptides between the HPV pair. The scale in this figure has been log-transformed. Each small square on the four panels depicts how many immunogenic peptides are shared between each pair of our selected viruses. Each heatmap represents one early protein (E1, E2, E6 and E7). For each protein, there were statistically significantly more peptide matches when comparing in-species HPV pairs to out-of-species HPV pairs. The statistical significance is preserved when the counts are added for all four proteins (see text for *p*-values).

After determining the overall protein homology, we investigated the conservation of HPV protein sequences at the peptide level. We used an in silico approach to identify HPV immunogenic peptides that could be presented to CD8^+^ T cells and trigger an immune response. We selected 18 of the most common HLA class I alleles for HLA-A, HLA-B, and HLA-C in the Caucasian population (Table S1). Using the NetMHCpan software (version 4.1) and 500 nM as the binding affinity threshold, we obtained a list of 9mer peptides that bind to the selected HLAs with the highest affinity. We defined a shared immunogenic peptide as one with at least 88% homology, in other words, with 8/9 identical amino acids in the same order [26]. HPV types within each species shared significantly more immunogenic peptides than HPV types that do not belong to the same species (*p*-value of 7.98 × 10^−99^, Figure 2). This holds true for each early protein: E1 (*p*-value of 1.37 × 10^−94^), E2 (*p*-value of 6.06 × 10^−95^), E6 (*p*-value of 3.64 × 10^−60^), and E7 (*p*-value of 8.73 × 10^−31^) (Table S4). As opposed to protein homology, immunogenic peptide homology was present to a much lesser degree at the genus level, especially for E6 and E7 (Figure 2). These findings indicate the potential cross-reactivity of T cells primed against antigens of certain HPV types to antigens from other HPV types in the same species.

### 3.3. Commensal HPVs Generate More Immunogenic Peptides Compared with α-HPVs

**Figure 3:**
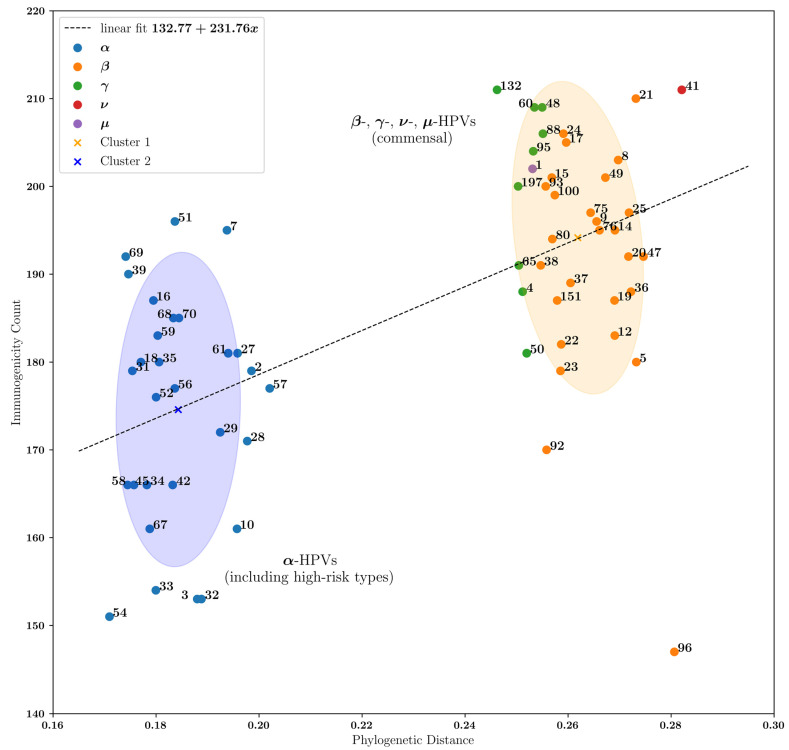
Relationship between HPV immunogenicity and phylogenetic distance. The *x*-axis represents the phylogenetic distance. We first used multiple sequence comparison by log-expectation (MUSCLE) as the multiple sequence alignment tool. We then used the standard procedure of first computing the distance using the percent identity homology and then using the neighbor joining tree construction algorithm, both available as a part of Biopython’s Phylo module. The *y*-axis represents the number of 9mer peptides that have a high binding affinity to the human leukocyte antigens (HLAs) selected in our study. This analysis was performed using the NetMHCpan software (version 4.1) and 500 nM as our binding affinity threshold. The high-risk carcinogenic α-HPVs, as defined by the NIH National Cancer Institute, include HPV 16, 18, 31, 33, 35, 39, 45, 51, 52, 56, 58, and 59. Two ellipsoids with centroid cluster centers were obtained by unsupervised gaussian mixture model clustering with two components and full covariance. There is a linear relationship of *y* = 231.76*x* + 132.77, with a statistically significant coefficient (*t*-statistic: 5.653, *p*-value < 0.001). Comparing α-HPVs versus other HPV genera yields a Mann–Whitney *U* test *p*-value of 1.31 × 10^−7^.

β-HPV evolution is largely unexplored but could give us important clues with regard to its potential for carcinogenesis, as well as its role in cutaneous immune homeostasis. As such, we sought to compare the immunogenicity of α-HPVs compared to other genera, including β, γ, μ, and ν, which are the etiologic agents of benign cutaneous warts. We obtained a list of immunogenic 9mer peptides as previously described and constructed a phylogenetic tree to determine the relationship between phylogenetic distance and immunogenicity between the HPV genera examined in this study. Statistically, when using a linear model and regressing the number of immunogenic peptides on the phylogenetic difference, we obtain a *p*-value < 0.001, demonstrating that the number of immunogenic peptides is dependent on the phylogenetic distance of the respective virus from a common ancestor. Our findings also show that HPV genera that are largely cutaneotropic—β, γ, μ, ν—have a larger phylogenetic distance from a common ancestor than α-HPVs, including the high-risk, carcinogenic HPVs. In addition to phylogenetic distance, these viruses cluster by the number of immunogenic peptides that they generate through in silico predictions of peptide–HLA binding affinities (Figure 3). Our findings suggest that commensal HPVs have evolved to generate more immunogenic peptides compared with α-HPVs (*p*-value of 1.31 × 10^−7^) (Table S5). It can also be observed that high-risk α-HPVs, including ones included in the current HPV vaccines, have a similar number of immunogenic peptides. However, the HPV vaccines use L1 capsid peptides for the purpose of generating neutralizing antibodies against infection with high-risk α-HPVs [27]. Interestingly, our findings reveal that novel T cell-directed immunotherapies may be effective in the treatment of cancers associated with certain high-risk α-HPVs (e.g., HPV 51) with a higher number of immunogenic peptides.

## 4. Discussion

Using an in silico approach, we report that early HPV protein homology translates to the inter-species conservation of immunogenic peptides that bind several HLA class I alleles with high affinity. We also show an evolution of cutaneotropic HPVs toward a more immunogenic phenotype. An evolutionary and epidemiological framework has been used by many in the field of HPV and has been argued to be crucial in the study of HPV functional differences and carcinogenic properties [28]. We propose that investigating immunogenicity at the peptide level across HPV genera using an evolutionary framework is essential to support functional studies in elucidating mechanisms of HPV immune evasion, carcinogenesis, and commensalism.

Cutaneous warts in OTRs represent a unique scenario that sheds light on the relationship between human host immunity and papillomaviruses in adults. In fact, OTRs develop immunosuppression later in life on an immunocompetent background for years, during which they have developed strong T cell responses to the HPV population colonizing their skin. T cells generate an immunodominance hierarchy wherein T cell immunity to some epitopes is stronger compared to others [29]. It can, therefore, be hypothesized that the specific HPV types found in the warts of OTRs overrepresent the epitopes for which this immunodominance has developed. Future research looking into the skin virome, especially the HPV diversity in the skin of OTRs is crucial. Our in silico data suggest that regardless of the variability in HPV types colonizing different individuals’ skin, the diversity of T cell clones recognizing HPV antigens is limited. This is an important concept when considering vaccine development against cutaneotropic HPVs to boost HPV-specific T cell responses which may protect patients from wart and cSCC development [7]. Our findings suggest that although the diversity of HPVs colonizing the skin is high, we may be able to achieve a broad anti-HPV protective immunity by immunizing individuals to a few representative peptides of the major HPV species found in the population. Our in silico-based approach using the NetMHCpan for peptide–HLA binding affinity determination has identified immunogenic peptides that have been verified to produce a cytotoxic CD8^+^ T cell response experimentally, increasing confidence in the validity and applicability of our findings [30].

In conclusion, our findings reveal that commensal HPVs may be more than quiet riders in our epithelial cells and may serve as immunogenic targets within the normal human epithelia that can be harnessed as therapeutic targets. Investigating host immunity to these viruses in human skin and mucosa is essential to understand their relationship with cancer development, especially in OTRs, as well as understanding the susceptibility of epidermodysplasia verruciformis and other immunodeficient patients to wart and cancer development.

## Data Availability

All data associated with the study are included in the manuscript. No additional data were created.

## Supplementary Materials

The following supporting information can be downloaded at: https://www.mdpi.com/article/10.3390/vaccines12070749/s1, Table S1. HLA class I alleles that are included in the study. Table S2. HPV types that are included in the study. Table S3. Summary statistics for the homology of in-species and out-of-species pairs of HPV viruses. Table S4. Summary statistics for the immunogenic peptide matches of in-species and out-of-species pairs of HPV viruses. Table S5. Summary statistics for the immunogenic peptide counts for alpha versus commensal (β, *γ*, μ, ν) HPV viruses. Table S6. HPV Sequences.
