# Supplementary material for: Commensal HPVs Have Evolved to Be More Immunogenic Compared with High-Risk α-HPVs
Source: Vaccines (Basel). 2024 Jul 7;12(7):749. doi: 10.3390/vaccines12070749 (PMC11281416; doi:10.3390/vaccines12070749)
Supplement: Supplementary file 1 [file vaccines-12-00749-s001.zip › vaccines-3030791-supplementary.pdf]

Supplementary Tables

Table S1. HLA class I alleles that are included in the study. EUR_freq: HLA allele frequency in the Caucasian population.

HLA-A	EUR_freq	HLA-B	EUR_freq	HLA-C	EUR_freq
HLA*02:01	0.29604	HLA*07:02	0.13987	HLA*07:01	0.16658
HLA*01:01	0.17181	HLA*08:01	0.12525	HLA*07:02	0.15006
HLA*03:01	0.14347	HLA*44:02	0.09011	HLA*04:01	0.10534
HLA*24:02	0.08686	HLA*15:01	0.06654	HLA*06:02	0.09301
HLA*11:01	0.05642	HLA*35:01	0.05713	HLA*05:01	0.09136
HLA*29:02	0.03279	HLA*40:01	0.05643	HLA*03:04	0.08215

Table S2. HPV types that are included in the study. Cutaneotropic HPV types found in common or recalcitrant warts of OTRs, as well as high-risk α-HPV types, were selected for this study.

Genus	Species	Type
Alpha (α)	1	HPV 32
		HPV 42
	2	HPV 3
		HPV 10
		HPV 28
		HPV 29
	3	HPV 61
	4	HPV 2
		HPV 27
		HPV 57
	5	HPV 51
		HPV 69
	6	HPV 56
	7	HPV 18
		HPV 39
		HPV 45
		HPV 59
		HPV 68
		HPV 70
	8	HPV 7
	9	HPV 16
		HPV 31
		HPV 33
		HPV 35
		HPV 52

		HPV 58<br>HPV 67
	11	HPV 34
	13	HPV 54
Beta (β)	1	HPV 5<br>HPV 8<br>HPV 12<br>HPV 14<br>HPV 19<br>HPV 20<br>HPV 21<br>HPV 24<br>HPV 25<br>HPV 36<br>HPV 47<br>HPV 93
	2	HPV 9<br>HPV 15<br>HPV 17<br>HPV 22<br>HPV 23<br>HPV 37<br>HPV 38<br>HPV 80<br>HPV 100<br>HPV 151
	3	HPV 49<br>HPV 75<br>HPV 76
	4	HPV 92
	5	HPV 96
Gamma (γ)	1	HPV 4<br>HPV 65<br>HPV 95
	2	HPV 48
	3	HPV 50
	4	HPV 60
	5	HPV 88
	12	HPV 132
	24	HPV 197
Mu (μ)		HPV 1
Nu (ν)		HPV 41

Table S3. Summary statistics for the homology of in-species and out-of-species pairs of HPV viruses. The p-values provided are obtained via a Mann–Whitney *U* (also known as Wilcoxon Rank-Sum) test.

Protein	Comparison	Mean	STD	Median	Min	Max	p-value
E1	in-species	0.7677	0.0928	0.7718	0.5838	0.9688	2.6e-99
	out-of-species	0.4611	0.0770	0.4277	0.3267	0.6637	
E2	in-species	0.6509	0.0908	0.6393	0.4665	0.8824	9.9e-100
	out-of-species	0.3495	0.0749	0.3228	0.232	0.5875	
E6	in-species	0.6642	0.1115	0.6454	0.4425	0.9433	1.4e-97
	out-of-species	0.3152	0.0869	0.2833	0.1834	0.6267	
E7	in-species	0.6876	0.1133	0.6923	0.4757	0.9307	1.8e-98
	out-of-species	0.3538	0.0803	0.3333	0.1892	0.6465	

Table S4. Summary statistics for the immunogenic peptide matches of in-species and out-of-species pairs of HPV viruses. The p-values provided are obtained via a Mann–Whitney *U* (also known as Wilcoxon Rank-Sum) test.

Protein	Comparison	Mean	STD	Median	Min	Max	p-value
E1	in-species	36.40	22.63	33.5	4	97	1.4e-94
	out-of-species	2.43	3.39	1	0	27	
E2	in-species	11.3293	8.6476	9	0	43	6.1e-95
	out-of-species	0.3762	0.9321	0	0	10	
E6	in-species	3.6402	3.8459	3	0	19	3.6e-60
	out-of-species	0.0234	0.178	0	0	3	
E7	in-species	1.6707	2.3272	1	0	13	8.7e-31
	out-of-species	0.0234	0.1607	0	0	2	
Total	in-species	53.04	34.17	47.5	10	153	8.0e-99
	out-of-species	2.85	4.03	1	0	27	

Table S5. Summary statistics for the immunogenic peptide counts for alpha versus commensal (β, γ, μ, ν) HPV viruses. The p-value provided is obtained via a Mann–Whitney *U* (also known as Wilcoxon Rank-Sum) test.

Immunogenic Peptides	Type	Mean	STD	Median	Min	Max	p-value
Total number per HPV type	alpha	174.59	12.66	177	151	196	1.3e-7
	commensal	194.16	12.58	195.5	147	211	
